# The impact of health coaching on the prevention of gestational diabetes in overweight/obese pregnant women: a quasi-experimental study

**DOI:** 10.1186/s12905-023-02750-0

**Published:** 2023-11-21

**Authors:** Fateme Mohammadian, Mouloud Agajani Delavar, Fereshteh Behmanesh, Alireza Azizi, Sedighe Esmaeilzadeh

**Affiliations:** 1 Department of Midwifery Counseling, School of Nursing and Midwifery, Fateme Mohammadian, Babol University of Medical Sciences, Babol, Iran; 2 Department of Midwifery, Infertility and Reproductive Health Research Center, Health Research Institute, Babol University of Medical Science, Babol, Iran; 3 Department of Psychiatry, School of Medicine, Babol University of Medical Sciences, Babol, Iran; 4 Department of Obstetrics and Gynecologist, Infertility and Reproductive Health Research Center, Health Research Institute, Babol University of Medical Science, Babol, Iran; 5 Infertility and Reproductive Health, Research Center of Babol, University of Medical Sciences, Babol-Amol old highway, after Mohammad hasan Khan bridge, Po. Box: 47135-547, Babol, Mazandaran, Iran

**Keywords:** Pregnancy, Overweight, Diabetes, gestational, Pregnant women, Mentoring

## Abstract

**Background:**

Previous studies have demonstrated that excessive gestational weight gain (GWG) increases the risk of gestational diabetes mellitus (GDM). This study aimed to determine the effect of using health coaching on the prevention of GDM in overweight pregnant women.

**Methods:**

In this quasi-experimental study, 64 eligible overweight women at 12–14 gestational weeks were randomly divided into 2 groups: the coaching group and the control group (usual care group). The intervention group received 8 weeks of the phone coaching program, which integrated GWG and physical activity to reduce the incidence of GDM. The Pregnancy Physical Activity Questionnaire (PPAQ) was used to assess physical activity during pregnancy. The occurrence of gestational diabetes was determined based on the 75-g 2-hour oral glucose tolerance test (OGTT) at 24–28 weeks of gestation. The primary outcome was the incidence of GDM, and the secondary outcomes included physical activity, GWG, and neonatal and maternal birth outcomes.

**Results:**

The incidence of GDM in the control and intervention groups was 24.1% and 22.6%, respectively. The relative risk (RR) was 0.93 (95% CI, 0.37–2.34; *P* = 0.887). The post survey results indicated that GWG decreased more considerably in the coaching than in the control group between pre-trial (T0) and post-trial (T1), (MD; -2.49 with 95% CI, -4.38 to -0.60; *P* < 0.011). Moreover, the total GWG (between pre-pregnancy and birth) diminished more remarkably in the coaching than in the control group, (MD; -2.83 with 95% CI, -5.08 to -0.58; *P* < 0.014). However, the score of self-efficacy and concern about PPAQ Metabolic Equivalent of Task (METs) did not differ between the coaching and control groups.

**Conclusions:**

The findings and implications of this research could significantly contribute to maternal health and gestational diabetes prevention. Additional support from a midwife coach resulted in better GWG. More studies are needed to assess the impact of health coaching as a component of usual care and its long-term effect on maternal and neonatal outcomes.

**Supplementary Information:**

The online version contains supplementary material available at 10.1186/s12905-023-02750-0.

## Background

Gestational diabetes mellitus (GDM) is a condition that involves impaired glucose and carbohydrate metabolism during pregnancy [1, 2]. Its prevalence has been reported as 7.9% in Iran [3]; it has increased by more than 30% in some developing countries during the last 2 decades [4, 5]. GDM is associated with adverse maternal and perinatal outcomes, including macrosomia, high blood pressure, increased cesarean section rate, preterm labor, shoulder dystocia, admission to the neonatal intensive care unit (NICU), and preeclampsia [6, 7]. In addition, it causes long-term complications, such as an increased risk of type 2 diabetes in the mother. Therefore, overweight and obesity are among the factors that can lead to gestational diabetes [8], which is strongly dependent on the excessive weight gain of mothers during pregnancy [9]. Indeed, the prevalence of GDM is increasing in parallel with overweight and obesity in the women [10]. Some health care professionals believe that excessive gestational weight gain (GWG) is a potential risk factor for GDM, especially in the first and second trimesters [11, 12]. In this regard, numerous studies have reported complications that can occur in mothers and fetuses both before and after birth [13, 14]. Adipose tissue accumulates in visceral depots more than in subcutaneous depots. Fat accumulation in visceral depots could increase the risk of developing insulin resistance and subsequent exhaustion of pancreatic b-cells, leading to inadequate insulin secretion and GDM [15, 16].

Nowadays, different interventions are used to prevent GDM, including lifestyle changes [17, 18], use of metformin [19], myo-inositol [20], dietary change [21], and physical activity [22, 23]. However, it can be challenging to implement these interventions during pregnancy. Therefore, it is preferable to use an easy, inexpensive, and safe intervention to prevent diabetes in pregnancy. Counseling (health coaching) has been shown to possess these qualities in some studies [24].

Health coaching is a unique approach that can widely improve health behaviors in patients with chronic conditions and is a tool to change unhealthy behaviors that lead to preventable diseases [25–27]. It is a combination of problem-solving, behavior modification, education, and psychosocial support [28]. So far, several studies have investigated the relationship between health coaching and GWG during pregnancy. In general, some of them concluded that health coaching had a positive effect on the prevention of excessive GWG [29]. In addition, Pamungkas et al. evaluated the effect of health coaching on the prevention of type 2 diabetes showed its positive effect alone or in combination with other innovations [30]. Our hypothesis is that pregnant women who receive midwife-led health coaching will have a positive impact on the prevention of GDM, lifestyle, and weight gain during pregnancy compared to those who receive conventional care. Therefore, this study aimed to assess the effect of midwife-led health coaching on GDM, GWG, physical activity, and pregnancy outcomes in women with a body mass index (BMI) of 25 kg/m^2^ or greater.

## Methods

This study was conducted between April 2022 and November 2022 at 2 obstetrics and gynecology clinics in Babol City, Iran. The Research Ethics Committee of Babol University of Medical Sciences approved this study (code: IR.MUBABOL.REC.1401.016).

Inclusion criteria were a pre-pregnancy BMI of 25 kg/m^2^ or greater, gestational age of 12–14 weeks, singleton pregnancy, and age over 18 years. Exclusion criteria were pre-pregnancy diabetes or fasting blood glucose of 92 mg/dL or greater at an initial prenatal appointment, prior GDM, family history of GDM, use of medications that can affect blood glucose (steroids, beta-adrenergic agonist, and antipsychotic drugs), physical disabilities, severe psychiatric disorder, and occurrence of vaginal bleeding or similar conditions that lead to the limitation of physical activity.

A sample size of 32 in each group was calculated based on, a 50% reduction in the incidence of gestational diabetes with significance level (alpha) of 0.05 (two-tailed) and 80% statistical power by G-power software [31].

**Fig. 1:**
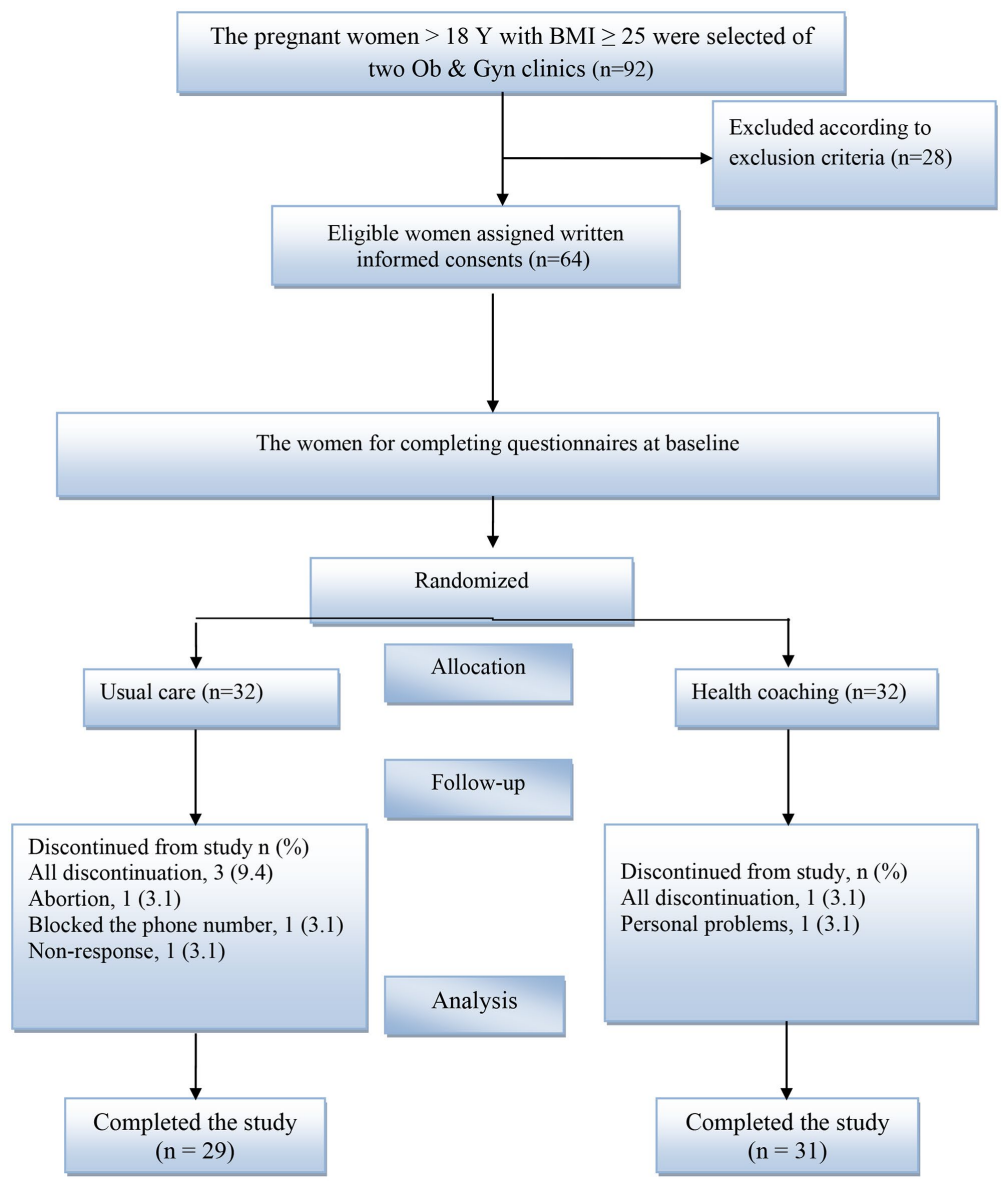
Consort fellow diagram of the participants

Of the 92 eligible pregnant women referred to the clinics, 28 were excluded according to exclusion criteria. In total, 64 women signed the written informed consent form. The women were randomly assigned to 2 groups: the usual care group and health coaching intervention group (1:1) according to the block of 4 using computer-generated random numbers. However, in a quasi-experimental design, true randomization may not be feasible due to various constraints and ethical considerations, but this study had the rationale for using a quasi-experimental design based on the existing literature, ethical considerations factors. In addition, due to the intervention methods of the 2 groups, blinding was not feasible, and the study was open-label. The dropout of the study was 6.3% (1 subject in the intervention group and 3 subjects in the control group; Fig. 1).

Both groups received standard prenatal care to ensure the safety of the mother and fetus, including risk assessment, physical examination, patient education, health promotion, and therapeutic intervention by 2 attending obstetricians. In the intervention group, in addition to standard prenatal care, 4 telephone call-based coaching sessions were conducted every 2 weeks (16–22 weeks of pregnancy), and each session lasted 30–45 min by a midwife coach (undergraduate midwife coach); however, it is important to note that standard prenatal care was not provided during the coaching sessions.

The health coaching protocol is based on the GROW (Goals, Reality, Options, and Will) coaching model to help pregnant women define their goals. In each session, the participant gave personal feedback on their nutrition and exercise habits during the last 5 days. Briefly, the goals of the coaching sessions include (1) promoting a positive attitude through education and skill development, (2) providing verbal and written access to evidence-based information about lifestyle (how to control weight and how to gain ideal weight during pregnancy and undertake at least 30 min of moderate regular exercise 3 to 4 days a week), (3) minimizing dangerous lifestyles and consume evidence-based healthy diets (such as fruits, high-fiber, whole-grain products, and vegetables), (4) optimizing weight control management, (5) problem-solving methods and activity planning, and (6) promoting active monitoring of weight gain during pregnancy and considering it important in preventing complications, including GWG.

All participants with a BMI of 30 kg/m^2^ or greater were advised to limit their GWG to 5 kg, in accordance with the guidelines set forth by the Institute of Medicine (IOM) [32]. To avoid complicating the intervention protocol, the same recommended rate of weight gain was used for those with a BMI of 29.0 to 29.9 kg/m2 (overweight). Throughout the rest of the pregnancy, the women’s weight gain was managed based on the guidelines established during the intervention period.

During the intervals between coaching sessions, the intervention group received short messages on various topics, such as recording weight changes, encouraging goal achievement, re-emphasizing positive lifestyle changes, and encouraging self-evaluation of the situation. Throughout the study, the participants were encouraged to identify obstacles to achieving their goals and to develop strategies to overcome them, with the help of the coaching program. This approach enabled them to make positive changes in their lifestyle and successfully achieve their weight management goals.

The primary outcome was the incidence of GDM for both groups. Diagnosis of GDM was made based on at least 1 abnormal value report of glucose 2 h after 75-g oral glucose tolerance test (OGTT) at 24–28 gestation weeks, and a fasting glucose (0 h) value ≥ 92 mg/dL, 1-hour ≥ 180 mg/dL, or 2-hour ≥ 153 mg/dL (2011) [33]. Secondary outcomes were pregnancy physical activity, GWG, and maternal and neonatal outcomes, including gestational age at delivery, cesarean section, birth weight, neonatal hospitalization rate, and preeclampsia.

Baseline socio-demographic and clinical characteristics were collected at enrollment using a checklist. Anthropometric measurements and physical activity levels of all participants were collected at both baseline and after the intervention period using the Pregnancy Physical Activity Questionnaire (PPAQ) and anthropometry measurements. Data on maternal and neonatal outcomes and birth weight were obtained from the medical records of the women. Finally, participants were asked about their experience with phone health coaching during pregnancy.

PPAQ is a standard self-reported questionnaire devised by Taber et al. for assessing physical activity of pregnant women [34]. In addition, the validity of its Persian version has been evaluated and confirmed by Abbasi et al. (2012). Its reliability was also determined by conducting a preliminary study on 20 eligible pregnant women with Cronbach’s alpha of 0.81 [35]. The Persian version questionnaire contains 32 questions about physical activities. PPAQ has 4 groups of questions, including questions related to household / caregiving (16 questions), transportation (3 questions), occupational activities (5 questions), and sports/exercise (9 questions). Each activity was grouped according to intensity into four categories namely; high (> 6.0 METs), moderate (3.0–6.0 METs), light (1.5–3.0 METs), and sedentary (1.5 METs). Furthermore, for each of the aforementioned levels of activity, the average number of METs per hour during the week was calculated. Activities were also classified according to their type (household/caregiving activities, occupational activi­ties, sports and exercise activities, transportation), giving the average number of METs per hour during the week spent on each type.

The participant’s weight was measured using a Seka scale (Germany) without shoes and with minimal clothing and an accuracy of 100 g. The measurements were taken at the initial antenatal visit (baseline) and again at 24 to 28 weeks. Height was measured using an inflexible tape measure mounted on the wall with an accuracy of 1 cm. Measurements were taken without shoes in a standing position with their heels attached to the wall and looking forward. The weight gain was changes in weight from baseline to 24–28 weeks gestation and also changes from baseline to birth. The body mass index was calculated using the formula of weight in kilograms divided by the square of the body height in meters, in accordance with the definition of the World Health Organization.

### Statistical analyses

SPSS version 22 (SPSS Inc., Chicago, IL., USA) was used for statistical analysis. The normality of data was tested using the Shapiro-Wilk W test, and continuous variables had a normal distribution. Descriptive statistics were used to describe continuous variables, and frequency distributions were used for categorical variables. To ensure the desirability of the random allocation process, the basic variables of the participants were checked in both groups after collecting the data in the first stage. The effect of health coaching during the follow-up period on GDM (each inappropriate glucose value) was expressed by calculating the relative risk (RR) at 95% CI. A multiple regression approach for analysis of covariance (ANCOVA) was used to assess the differences in physical activity and GWG between the pre-trial and post-trial phases in the 2 groups with controlling the effect of variables such as age, pre-pregnancy weight, and pre-pregnancy BMI as a covariate.

Also, we used chi-square and Student *t* tests for differences in continuous and categorical variables between the 2 groups. *P* values less than 0.05 were considered statistically significant.

## Results

All baseline characteristics (including age, education level, occupation, residence, family income, pre-pregnancy weight, pre-pregnancy BMI, primipara, history of abortion, and history of cesarean section) were similar in both groups (Table 1).

**Table 1:** Baseline socio-demographic and clinical characteristics of the participants in the control and Coaching groups

Variables	Control group (n = 29)	Coaching group (n = 31)	P-value	
**Age (years)**				
< 30	13 (44.8)	15 (48.4)	0.782	
≥ 30	16 (55.2)	16 (51.6)		
**Education**				
Primary/ secondary education	17 (58.6)	20 (64.5)	0.639	
Higher education	12 (41.4)	11 (35.5)		
**Occupation**				
Housewife	20 (69.0)	25 (80.6)	0.296	
Work out	9 (31.0)	6 (19.4)		
**Residence**				
Urban	20 (69.0)	24 (77.4)	0.459	
Rural	9 (31.0)	7 (22.6)		
**Family income (Rials)**				
< 5 million	11 (37.9)	10 (32.3)	0.645	
≥ 5 millions	18 (62.1)	21 (67.7)		
**Gestational age, Mean (SD)**	12.9 (0.8)	13.2 (0.8)	0.196	
**Primipara**	9 (31.0)	7 (22.6)	0.459	
**History of abortion**	9 (31.0)	12 (38.7)	0.533	
**History of section**	12 (70.6)	14 (70.0)	0.699	

The incidence of GDM in the control and intervention groups was 7/29 (24.1%) and 7/31 (22.6%), respectively. The relative risk was 0.93 (95% CI, 0.37–2.34; *P* = 0.887) (Table 2).

**Table 2:** Comparison of gestational diabetes in 24–28 weeks of pregnancy of the participants in the control and Coaching groups

Variables	Control group (n = 29) N (%)	Coaching group (n = 31) N (%)	RR (95% CI)	P-value
Gestational diabetes*	7 (24.1)	7 (22.6)	0.93 (0.37 to 2.34)	0.887
Fasting blood glucose ≥ 92 mg/dL	5 (17.2)	5 (16.1)	0.94 (0.29 to 2.97)	0.922
Blood glucose 1-hour ≥ 180 mg/dL	3 (10.3)	3 (9.7)	0.94 (0.24 to 4.33)	0.938
Blood glucose 2-hour ≥ 153 mg/dL	1 (3.4)	3 (9.7)	2.65 (0.29 to 24.11)	0.388

*Diagnosis according to glucose 2 h after 75 gram oral glucose (OGTT) at 24–28 gestation weeks

The post survey results indicated that GWG decreased more considerably in the coaching than in the control group between pre-trial (T0) and post-trial (T1), (MD; -2.49 with 95% CI, -4.38 to -0.60; *P* < 0.011). Moreover, the total GWG (between pre-pregnancy and birth) diminished more remarkably in the coaching than in the control group, (MD; -2.83 with 95% CI, -5.08 to -0.58; *P* < 0.014). The physical activity of participants in both groups was recorded before and at 24–28 weeks. The score of self-efficacy and concern about PPAQ Metabolic Equivalent of Task (METs) did not differ between the coaching and control groups. The sports/exercise METs increased in the coaching group, but the difference was not statistically significant (Table 3).

**Table 3:** Comparisons of weight and physical activity in of the participants in the control and Coaching groups

	(1) Control group (n = 29)		(2) Coaching group (n = 31)		Mean Difference (2 − 1)	P-value^d^
	T0 Mean (SD)	T1 Mean (SD)	T0 Mean (SD)	T1 Mean (SD)		
Weight at 24–28 weeks (Kg)	79.3 (13.9)	84.8 (0.7)	75.6 (9.0)	82.3 (0.7)	-2.49 (-4.38 to -0.60)	0.011
Weight at end-pregnancy (Kg)	79.3 (13.9)	90.4 (0.8)	75.6 (9.0)	87.5 (0.8)	-2.83 (-5.08 to -0.58)	0.014
Total PPAQ^a^ (MET-h/wk)^b^	86.0 (49.0)	97.5 (43.6)	78.2 (33.6)	90.0 (38.5)	-9.02 (-45.92 to 27.89)	0.403
**By intensity (MET-h/wk)**						
Sedentary (< 1.5 METs)	43.4 (27.9)	45.9 (28.8)	48.9 (27.5)	34.9 (20.9)	-8.83 (-19.26 to 1.60)	0.091
Light-intensity activity (1.5 - <3.0 METs)	38.2 (26.9)	35.0 (20.4)	29.0 (14.1)	36.9 (15.2)	-0.59 (-6.40 to 5.22)	0.822
Moderate-intensity activity(3.0–6.0 METs)	24.1 (19.4)	16.1 (12.7)	16.0 (15.9)	16.9 (14.8)	2.19 (-4.51 to 0.90)	0.478
Vigorous-intensity activity (> 6.0 METs)	1.1 (1.9)	0.4 (0.8)	0.8 (1.7)	0.9 (1.8)	1.14 (-0.22 to 2.51)	0.098
**By type (MET-h/wk)**						
Household / caregiving	50.9 (36.1)	41.0 (22.6)	43.7 (29.6)	48.4 (25.5)	3.71 (-13.1 to 20.5)	0.595
Occupational activity	14.2 (25.0)	7.7 (18.6)	7.1 (18.9)	4.9 (14.1)	-2.82 (-10.88 to 5.23)	0.484
Sports/exercise	1.7 (2.4)	1.4 (2.3)	0.7 (1.0)	1.5 (2.4)	0.41 (-0.40 to 1.23)	0.312
Transportation	8.2 (8.0)	7.8 (6.9)	4.5 (5.7)	5.4 (5.1)	-1.21 (-4.27 to 1.86)	0.434

^a^ PPAQ: Pregnancy Physical Activity Questionnaire; ^b^MET-h•wk: MET metabolic equivalent turnover ; ^c^ Among participants who were currently work in work out (25% of the subjects)

^d^ covariate variables: age, pre-pregnancy weight and pre-pregnancy BMI

In addition, the mean total pregnancy weight gain in the control and intervention groups was 12.7 ± 5.4 and 10.2 ± 3.4 kg, respectively (*P* = 0.036). The mean weight gain between pre-pregnancy and 24–28 weeks in the control and intervention groups was 7.2 ± 3.2 and 5.1 ± 4.2 kg, respectively (*P* = 0.038). There was no significant difference in birth weight, gestational age at delivery, cesarean section, neonatal hospitalization rate, and preeclampsia between the coaching and control groups. However, the percentage of hospitalized neonates was 31.0% in the control group and 16.1% in the coaching group. Furthermore, in the control group, 2 patients had preeclampsia, while in the intervention group, no preeclampsia was observed (Table 4).

**Table 4:** Maternal and neonatal pregnancy outcomes of the participants in the control and Coaching groups

Variables	Control group (n = 29) Mean (SD)	Coaching group (n = 31) Mean (SD)	P-value
Weight gain by 24–28 weeks	7.2 (3.2)	5.1 (4.2)	0.038
Total weight gain by delivery	12.7 (5.4)	10.2 (3.4)	0.036
Birth weight (gr)	3293.1 (476.3)	3393.7 (318.6)	0.344
Gestational age at birth (weeks)	38.3 (1.9)	38.7 (1.0)	0.345
Caesarean section, n (%)	20 (69.0)	19 (61.3)	0.533
Neonatal hospitalization rate, n (%)	9 (31.0)	5 (16.1)	0.173
Preeclampsia, n (%)	2 (6.9)	0 (0.0)	0.229

## Discussion

This quasi–experimental study investigated the effectiveness of an 8-week phone coaching program, which integrated GWG and physical activity, in reducing the incidence of GDM in overweight or obese pregnant women. The program was delivered by a midwife coach through 4 telephone sessions, each lasting 30–45 min. However, the study found that the program did not result in a significant reduction in the incidence of GDM when compared to the control group. However, the intervention group reported high satisfaction with phone health coaching during pregnancy. This intervention focused on promoting a healthy diet during pregnancy, GWG, and moderate regular exercise throughout pregnancy to prevent GDM. Most published studies used the health coaching intervention to control and treat type 2 diabetes and GDM [29, 30, 36, 37].

Our findings are consistent with those of a meta-analysis that included 23 studies with a total of 8,877 overweight or obese women aimed to prevent GDM. According to this meta-analysis, the physical activity and/or diet interventions (alone or in combination) were less effective than placebo in reducing the risk of GDM [38]. In contrast, our study showed that the coaching intervention was an effective intervention to improve GWG. However, improved GWG in overweight/obese women was not associated with the prevention of GDM or improved maternal and neonatal outcomes.

There are several potential explanations for the lack of significant effects of our intervention on the prevention of GDM among overweight or obese women. A meta-analysis [39] reported that physical exercise during pregnancy was associated with a lower risk of GDM (31% reduction). However, our study showed that health coaching could not improve the physical activity level of the participants during pregnancy. This may be due to the interference time of our study with the COVID-19 pandemic. In this regard, one of the main challenges faced by the intervention group was the restrictions on sports activities during pregnancy and the closure of childbirth preparation classes. Also, during this study, 25.8% of participants in the intervention group and 20.7% in the control group were diagnosed with COVID-19. Finally, it is recommended that an intervention should be performed during the first and second trimesters of the pregnancy [40]. While in our study, the intervention was performed during the second trimester of pregnancy. Further large clinical trials are required to investigate the efficacy of coaching interventions focused on the first trimester of pregnancy in overweight or obese pregnant women.

In our study, maternal and neonatal outcomes were desirable in both groups, with no adverse outcomes. Only 2 cases of low birth weight and preterm labor were observed in the control group. In both groups, the cesarean delivery rate was almost equal. In the intervention group, there were 2 cases of normal vaginal birth after cesarean section. A Cochrane review of 15 trials found no evidence of a difference between the control and lifestyle intervention groups in maternal outcomes, such as cesarean section and preeclampsia in treating women with GDM [41]. However, in a 2021 study that aimed to investigate the effectiveness of health coaching in managing gestational diabetes among women with GDM, the newborn outcomes showed improvement, but the maternal outcomes in the intervention group were not favorable [42].

There are some limitations in this study. First, this study used a non-probability sampling method, while future studies that use random sampling can provide stronger evidence on this intervention. Second, this study had a single-center design with a short intervention. Third, risk factors of gestational diabetes are varied so the risk factors can affect the incidence of gestational diabetes in our participants; however we considered the gestational diabetes risk factors in our exclusion criteria and excluded women with a higher risk of gestational diabetes. We suggest future studies consider having polycystic ovary syndrome of participants and dietary pattern of pregnant women. Moreover, further studies with longer interventions that include both the first and second trimesters of pregnancy can provide more relevant results. According to a meta-analysis, the prevention of GDM in overweight or obese pregnant women may require interventions that target both the first and second trimesters of pregnancy. Third, we did not assess compliance with a healthy dietary pattern, which will impact the association of the coaching intervention with GDM; however, our coaching intervention aimed to promote a healthy diet that supports both GWG and the prevention of GDM, as this was a key goal of the coaching sessions. Finally, participants in the control group might have taken information on physical activity and control weight from the participants in the intervention group, which might cause a confirmation bias.

## Conclusions

Despite the limitations, the findings implications of this research could significantly contribute to maternal health and gestational diabetes prevention. We showed that additional support from a midwife coach resulted in better GWG, but it did not affect the prevention of GDM. Considering that a few studies have been conducted to investigate the effect of health coaching on GDM, it is recommended to assess the impact of health coaching as a component of standard prenatal care, as well as to assess GDM and its long-term effects on maternal and neonatal outcomes.

### Electronic supplementary material

Below is the link to the electronic supplementary material.

Supplementary Material 1

Supplementary Material 2

## Data Availability

All data generated or analyzed during this study are included from preliminary studies are available from the corresponding author on reasonable request.

## List of abbreviations

GWG: Gestational weight gain
GDM: Gestational diabetes mellitus
PPAQ: Pregnancy Physical Activity Questionnaire
OGTT: Oral glucose tolerance test
RR: Rrelative risk
NICU: Neonatal intensive care unit
BMI: Body mass index
I IOM: Institute of Medicine
ANCOVA: analysis of covariance
