# Supplementary material for: The impact of health coaching on the prevention of gestational diabetes in overweight/obese pregnant women: a quasi-experimental study
Source: BMC Womens Health. 2023 Nov 21;23:619. doi: 10.1186/s12905-023-02750-0 (PMC10664614; doi:10.1186/s12905-023-02750-0)
Supplement: Supplementary file 1 — Supplementary Material 1 [file 12905_2023_2750_MOESM1_ESM.pdf]

EDITORIAL CERTIFICATE

This document certifies that the manuscript listed below was edited for proper English language, grammar, punctuation, spelling, and overall style by one or more of the highly qualified native English speaking editors at NedMedica

Manuscript title:

The Impact of Health Coaching on the Prevention of Gestational Diabetes in Overweight/Obese Pregnant Women: A Quasi-Experimental Study

Authors:

Mouloud Agajani Delavar

Date Issued:

June 26, 2023

Editing Certified
CERTIFICATE

NedMedica®

Dr. Seyyed Mohammad Miri
Managing Director

Central Office: Kerkraderweg 13, 6416 CC, Heerlen, The Netherland Tel: +31615314198 | Fax: +31452051123
website: www.Nedmedica.nl | Email: Info@Nedmedica.nl
